# Abnormal coronary vascular response in patients with long COVID syndrome – a case‐control study using oxygenation‐sensitive cardiovascular magnetic resonance

**DOI:** 10.1016/j.jocmr.2025.101890

**Published:** 2025-04-02

**Authors:** Lukas D. Weberling, Elizabeth Hillier, Matthias G. Friedrich, Marc Zahlten, Norbert Frey, Florian André, Henning Steen

**Affiliations:** a Department of Cardiology, Angiology and Pneumology, Heidelberg University Hospital, Heidelberg, Germany; b DZHK (German Centre for Cardiovascular Research), Partner Site Heidelberg/Mannheim, Heidelberg, Germany; c Faculty of Medicine and Health Sciences, Division of Experimental Medicine, McGill University, Montreal, Quebec, Canada; d Division of Cardiology, Departments of Medicine and Diagnostic Radiology, McGill University, Montreal, Quebec, Canada

**Keywords:** CMR, Cardiovascular imaging, OS-CMR, Oxygenation-sensitive CMR, COVID-19, Long COVID syndrome

## Abstract

**Background:**

Following the worldwide coronavirus disease 2019 (COVID-19) pandemic, many patients reported ongoing severe cardiovascular symptoms after the acute phase. This multisystemic condition has been named long COVID syndrome. While cardiovascular magnetic resonance (CMR) imaging is the gold standard to diagnose acute myocardial damage, no specific changes have been shown in long COVID patients. However, endothelial dysfunction has been hypothesized to contribute to its pathogenesis. Oxygenation-sensitive CMR during breathing exercise is a simple, non-invasive, and accurate test to objectify vascular function, which has not been applied to long COVID patients yet.

**Methods:**

After receiving approval from the local ethics committee, this prospective observational case-control study enrolled (i) patients reporting symptoms for ≥6 weeks following an acute COVID-19 infection or vaccination, and (ii) healthy volunteers with neither symptoms nor history of cardiovascular disease. Participants completed a questionnaire, point-of-care testing of cardiac biomarkers, a standard non-contrast CMR, and an oxygenation-sensitive CMR. Heart rate response and breathing-induced myocardial oxygenation reserve (B-MORE) were assessed during metronome-paced hyperventilation and apnea.

**Results:**

Thirty-one patients (17 female; age 39.4 [30.3; 51.6] years) and 27 controls (12 female; age 33.3 [27.3; 46.8] years) were included with comparable demographics and cardiovascular risk factors between groups. Laboratory testing and standard CMR did not reveal any pathologies in either of the groups. Indexed left ventricular stroke volume was significantly lower in patients (44.5 mL [41.2; 46.6] vs 55.9 mL [49.2; 59.2]; p < 0.001), while ejection fraction and longitudinal strain of both ventricles were comparable (p > 0.05 for all). Vasoactive breathing exercises induced a significant increase in heart rate (+35/min [21; 45]) and B-MORE (9.8% [4.3; 17.2]) in controls. In patients, however, heart rate increase was blunted (+15/min [7; 26]; p < 0.001) and B-MORE was significantly lower (7.3% [3.4; 10.4], p = 0.044).

**Conclusion:**

This pilot study is the first to show a blunted hemodynamic and myocardial oxygenation response to vasoactive breathing maneuvers during oxygenation-sensitive CMR in long COVID patients. This simple, non-invasive test may be the first to objectify complaints of affected patients and indicates evidence for the crucial role of the endothelium in the pathophysiology of long COVID.

## Graphical abstract

**Figure:**
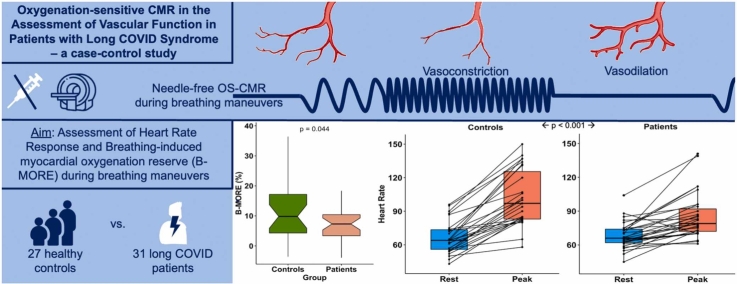

## 1 Background

The unprecedented spread of the coronavirus disease 2019 (COVID-19) has challenged the limits of global health care systems with currently over 775 million cases and over 7 million deaths [1]. COVID-19 is a multisystemic disease affecting respiratory and in particular cardiovascular systems [2]. Affected patients experience different clinical courses ranging from asymptomatic infections to a potentially life-threatening illness, with the latter occurring less common since the introduction of COVID-19 vaccinations [3]. In a minority of patients, symptoms persist >4 or >12 weeks after the acute phase of infection with shortness of breath, fatigue, chest pain, brain fog, and myalgia being the most common complaints. This symptom complex has been summarized as long COVID syndrome and may also, to a much lesser extent, occur after COVID-19 vaccination [4], [5]. It also occurs in initially asymptomatic or mild infections and does not correlate with myocardial injury assessed by cardiovascular magnetic resonance (CMR) imaging [6], [7]. Furthermore, vaccination has been shown to only partly protect from long COVID syndrome [8]. Therefore, despite a high individual symptom burden and decreased quality of life, it has proven challenging to identify underlying causes and to provide potential targets for therapies. Suggested mechanisms involve persisting tissue damage or virus particles, coagulopathies, and metabolic or autoimmune disorders [9], [10]. Recent research indicates a crucial role of the endothelium in the pathogenesis of the disease [9], [11], [12], [13].

In the assessment of the cardiovascular autonomic and endothelial function, combining oxygenation-sensitive CMR (OS-CMR) with breathing maneuvers is highly reproducible and has been established in patients with coronary artery disease, obstructive sleep apnea, myocardial infarction with non-obstructive coronary arteries, heart failure and in heart transplant recipients [14], [15], [16], [17], [18], [19], [20]. Hyperventilation is a stressor superior to adenosine and substantially increases heart rate, specifically in healthy individuals [21]. OS-CMR makes use of the different relaxation of oxygenated versus deoxygenated hemoglobin measurable through signal alterations in the highly perfused myocardium [15]. In a standardized breathing sequence including hyperventilation followed by a breath-hold, CO_2_ induces a coronary vasoconstriction followed by a vasodilation [20]. These endothelium-dependent breathing effects induce specific changes of oxygenation in the myocardium, which can be assessed through OS-CMR and which have been standardized as breathing-induced myocardial oxygenation reserve (B-MORE) [22].

The aim of this study was to assess the myocardial vascular and autonomous function in long COVID patients using OS-CMR during standardized breathing maneuvers.

## 2 Methods

### 2.1 Study population and design

**Fig. 1:**
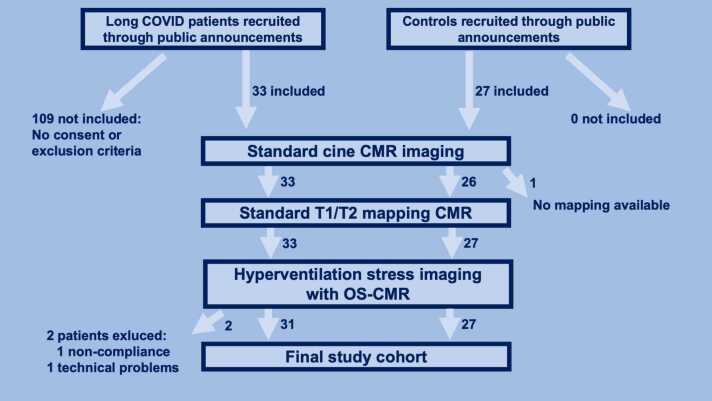
Flowchart of recruitment, inclusion, and examination of both long COVID cases and healthy controls. *COVID* coronavirus disease, *CMR* cardiovascular magnetic resonance, *OS-CMR* oxygenation-sensitive cardiovascular magnetic resonance

This prospective observational case-control study was approved by the ethics committee of the University of Heidelberg, Germany (S-361/2022). Participants were included through public announcements and social media posts. A full written informed consent was obtained from all participants. Between September 2022 and January 2024, two groups of participants (patients and controls) were prospectively included at two participating sites in Hamburg and Hannover, Germany. For the patients, inclusion criteria were either [1] the persistence of symptoms, [2] the new onset of symptoms, or [3] a worsening of symptoms with a significant negative influence on daily activities and quality of life for at least 6 weeks after an acute COVID-19 illness (with positive polymerase chain reaction or rapid antigen testing) or a COVID-19 vaccination in accordance to the long/post-COVID guidelines [23]. A group of healthy volunteers without the presence of any cardiovascular symptoms but with a history of COVID-19 vaccination or infection at least 6 weeks before inclusion served as controls. Exclusion criteria for both groups involved known cardiac pathologies (such as heart failure, coronary artery disease) or a COVID-19 infection or vaccination in the last 6 weeks before the examination. Of note, participants with cardiovascular risk factors or isolated atrial fibrillation were not excluded. Fig. 1 shows a flow diagram of the patient recruitment. The study involved a questionnaire covering pre-existing conditions, cardiovascular risk factors, and current complaints, a medical consultation (in person or via telephone), point-of-care testing of cardiac biomarkers (high-sensitivity troponin I [hsTnI] and N-terminal pro-hormone brain natriuretic peptide [NTproBNP], Quidel Triage, San Diego, California, USA), a standard CMR and a vascular function assessment using breathing maneuvers with OS-CMR.

### 2.2 CMR acquisition protocol

**Fig. 2:**
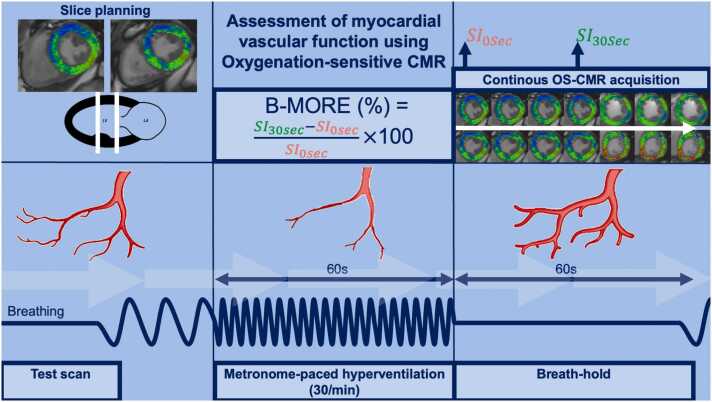
Study protocol to assess myocardial vascular function using oxygenation-sensitive CMR. *B-MORE* breathing-induced myocardial oxygenation reserve, *SI* signal intensity, *CMR* cardiovascular magnetic resonance, *OS-CMR* oxygenation-sensitive cardiovascular magnetic resonance

CMR acquisitions were executed on clinical CMR scanners of the manufacturer Siemens Healthineers (Erlangen, Germany) at either 1.5T (site in Hannover, Magnetom™ Aera) or 3T (site in Hamburg, Magnetom™ Skyra). Cine images were obtained with a steady-state free precession sequence (time of repetition 38.4 ms; time of echo 1.24–1.4 ms; field of view 301 × 360/174 × 208 mm; slice thickness 8.0 mm) using retrospective electrocardiographic gating in at least three long-axis planes (two-, three-, and four-chamber views), as well as a short-axis stack of the whole left ventricle (LV) (during breath-hold with 25 phases per cardiac cycle). T1 maps were acquired using a standard modified look-locker inversion recovery 5s(3s)3s T1-native sequence in 3–5 short-axis views (time of repetition 280.6–312.6 ms; time of echo 1.12–1.33 ms; field of view 360 × 306/384 × 327 mm; slice thickness 8.0 mm). T2 maps were acquired using a T2 FLASH sequence in 3–5 short-axis views (time of repetition 222.8–280.6 ms; time of echo 1.12 ms; field of view 360 × 306/384 × 327 mm; slice thickness 8.0 mm). OS-CMR was executed using a modified balanced steady-state free precession sequence (time of repetition 40.7 ms; time of echo 1.7 ms; field of view 320 × 380/162 × 192 ms; slice thickness 10 mm) in two short-axis slices (basal and midventricular) as previously described [15], [18]. The standard OS-CMR imaging protocol assessing vascular function was deployed as previously described and involved a baseline OS-CMR image acquisition, followed by 60 s of metronome-paced hyperventilation at 30 breaths/min, and then by a voluntary maximal breath-hold at expiration [15], [18]. OS-CMR images were acquired continuously during breath-hold. Oxygen saturation, blood pressure, and heart rate were noted before initiation of hyperventilation. Participants received a minimum of three repetitions of exact instructions for the breathing maneuvers (with the first one through a standardized video) and did at least one test hyperventilation before the scan. During hyperventilation, the maximum heart rate measured by electrocardiogram and pulse oximeter was noted. Heart rate response was calculated by subtracting the baseline heart rate from the maximum heart rate. Patient compliance, breathing depth, and breathing rate were monitored visually and with a respiration belt and rated as excellent, good, acceptable, or poor. Side effects experienced by the patients were noted. Fig. 2 illustrates the OS-CMR protocol. No contrast agent was administered during the CMR acquisition.

### 2.3 Image analysis

Image analysis was performed using cvi42 (versions 5.13.8 and 6.0.2, Circle Cardiovascular Imaging, Calgary, Alberta, Canada), including the research module OxySense by a researcher (L.D.W.) with >4 years and >1500 clinical CMR cases of experience, who was blinded to all clinical data. Endocardial and epicardial borders of both the LV and right ventricle (RV), the left and right atrium (LA, RA), and both RV insertion points throughout the cardiac cycle were automatically detected and manually corrected, if necessary, to derive volume, myocardial mass, ejection fraction (EF) and longitudinal strain of both ventricles and atria and to measure global T1 and T2 values. Global oxygenation (B-MORE) was measured at the end of hyperventilation (0 s, at the beginning of breath-hold) and at 30 s during the breath-hold. For these measurements, end-systolic images of each cardiac cycle were identified and endocardial and epicardial contours were drawn using the automated processing tool of the OxySense module, which were manually corrected if needed. The global B-MORE was calculated by the formula B-MORE (%) = $\frac{{\mathrm{SI}}_{30\sec}-{\mathrm{SI}}_{0\sec}}{{\mathrm{SI}}_{0\sec}}\times 100$.

### 2.4 Statistical analysis

The R language and environment for statistical computing (version 4.2.1, R Foundation for Statistical Computation, Vienna, Austria) with the R studio user interface (version 2023.06.0+421) was deployed for the statistical analyses [24].

Normal distribution was assessed by using the Shapiro-Wilk test. Parametric variables are given as mean ± standard deviation, nonparametric variables as median with interquartile range. The Student’s t-test was used for the comparison of two groups, when parameters were distributed normally. The nonparametric Wilcoxon rank-sum test tested not normal distributed variables for differences. The Fisher’s exact test was used to test for group differences of categorical variables. Potential correlations between two variables were assessed using the Pearson or Spearman’s rank correlation coefficient, as applicable. To test the influence of the potential confounders sex, age, hypertension, and asthma, an analysis of covariance was used.

## 3 Results

### 3.1 Study population

Fifty-eight participants (27 controls; 31 patients) were included in this study. Both patients and controls were at a young age and distribution among genders was balanced. The presence of cardiovascular risk factors was scarce with hypertension and a smoking habit being most frequent in both patients and controls. None of the controls and 14 of the patients had known comorbidities. These involved a thyroid disorder (3), asthma (3), celiac disease (2), iron deficiency, polycystic ovary syndrome, testosterone deficiency, prior Lime disease, prior pulmonary embolism, atrial fibrillation, and obstructive sleep apnea (1 each). Only a fraction of both controls and patients were on a vasoactive medication. Five controls were non-professional endurance athletes, while the rest of the controls had ordinary levels of physical activity (<5 h/week). Baseline characteristics are given in Table 1.

**Table 1:** Baseline characteristics of cases and controls.

	Controls		Long COVID	
	n = 27		n = 31	
Age [years]	33.3	[27.3; 46.8]	39.4	[30.3; 51.6]
Body mass index [kg/m²]	24.4	[21.9; 29.2]	24.9	[21.0; 27.3]
Sex [male/female]	15	12	14	17
Cardiovascular risk factors				
Hypertension	7	25.9%	7	22.5%
Hypercholesterinemia	5	18.5%	6	19.4%
Diabetes mellitus	0	0.0%	0	0.0%
Smoking habit	6	22.2%	8	25.8%
Family history	0	0.0%	4	12.9%
On vasoactive medication	1	3.7%	6	19.4%
Calcium antagonists	0	0.0%	2	6.5%
Beta blocker	1	3.7%	4	12.9%
Laboratory testing				
hsTnI [ng/L]	2.5*	[1.6; 3.5]	1.2	[0.3; 2.5]
NTproBNP [ng/L]	24*	[20; 45]	24	[20; 67]

*COVID* coronavirus disease, *NTproBNP* N-terminal pro-hormone brain natriuretic peptide, *hsTnI* high-sensitivity troponin I

Continous values are given as median (Interquartile range). Binary values are given as percentages with numerator and denominator.

* Laboratory testing was available for 8 healthy controls

Patients were highly symptomatic at the time of enrollment with angina pectoris, dyspnea, and fatigue being the most common complaints. Brain fog, myalgia, anxiety, or depression were also common among patients. The prevalence of symptoms was similar between patients who developed long COVID following infection versus vaccination. Details of the present complaints are displayed in Table 2. The laboratory testing revealed normal levels of hsTnI and NTproBNP in all study participants.

**Table 2:** Present complaints of the long COVID groups.

	All cases		Post COVID-19		Post vaccination		Group difference
	n = 31		n = 14		n = 17		p =
Angina pectoris	**27**	**87.1%**	**11**	**78.6%**	**16**	**94.1%**	0.607
Typical	8	25.8%	3	21.4%	5	29.4%	
Atypical	19	61.3%	8	57.1%	11	64.7%	
Dyspnea	**21**	67.7%	**9**	**64.3%**	**12**	**70.6%**	0.814
On exertion	13	41.9%	5	35.7%	8	47.1%	
At rest and on exertion	8	25.8%	4	28.6%	4	23.5%	
Brain fog	20	64.5%	8	57.1%	12	70.6%	0.478
Myalgia	19	61.3%	8	57.1%	11	64.7%	0.724
Fatigue	24	77.4%	10	71.4%	14	82.4%	0.670
Anxiety or depression	7	22.6%	5	35.7%	2	11.8%	0.198
Duration of complaints [d]	559	[277; 663]	227	[125;599]	600	[555; 769]	**0.018**

*COVID-19* coronavirus disease 2019

Significant group differences are highlighted in bold.

Continous values are given as median (Interquartile range). Binary values are given as percentages with numerator and denominator.

### 3.2 Standard CMR

For the CMR scan, 51 participants were scanned at 3T, while 7 participants (4 controls, 3 patients) were scanned at 1.5T. There were no wall motion abnormalities in any of the participants. LV- and RV-EF were normal in both controls (median LV-EF 61.0%; RV-EF 56.0%) and patients (LV-EF 59.7%; RV-EF 55.4%) and were similar between groups. Equally, left and right global longitudinal strain (GLS) were normal in both controls (median LV-GLS −19.4%; RV-GLS −24.0%) and patients (−18.5%; −22.7%) and were comparable between groups. Controls had higher volumes (indexed to body surface area) of both the LV (controls 89.7 mL; patients 72.9 mL) and the RV (96.7 mL; 78.3 mL) and showed higher indexed stroke volumes of both the LV (55.9 mL vs 44.5 mL) and the RV (53.7 mL vs 45.6 mL). This difference remained significant even after excluding athletic controls (n = 5) from the control group. The maximum wall thickness of the LV was normal and similar between controls (median 9 mm) and patients (8 mm). Global T1 and T2 times were all within scanner-specific normal value ranges and values at 3T were comparable between controls (median T1 1232 ms; median T2 41 ms) and patients (T1 1232 ms, T2 40 ms). T1 values at 1.5T showed a significant difference (p = 0.042) between cases (1009 ms) and controls (1092 ms), which is however underpowered due to the small sample size at 1.5T (4 controls, 3 patients) and was not significant after adjustment for confounders. The adjustment for the confounders age, sex, hypertension, and asthma for all other previous analyses did not alter the results. Table 3 shows the CMR results of both groups and both unadjusted and adjusted group differences.

**Table 3:** Results derived from the standard contrast-free CMR in cases and controls.

						Group difference	
		Controls		Long COVID		Unadjusted	Adjusted*
		n = 27		n = 31		p =	p =
LV-EDV (indexed, mL/m²)		89.7	[83.1; 95.2]	72.9	[69.1; 83.9]	**0.001**	**<0.001**
LV-ESV (indexed, mL/m²)		34.8	[31.1; 38.8]	30.5	[25.9; 34.8]	0.053	**0.019**
LV-EF (%)		61.0	[57.5; 64.0]	59.7	[56.1; 62.6]	0.384	0.350
LV-GLS (-%)		19.4	[17.7; 20.6]	18.5	[17.7; 20.0]	0.647	0.616
LV-SV (indexed, mL/m²)		55.9	[49.2; 59.2]	44.5	[41.2; 46.6]	**<0.001**	**<0.001**
Max. wall thickness LV		9	[8; 11]	8	[8; 11]	0.541	0.198
RV-EDV (indexed, mL/m²)		96.7	[88.5; 102.3]	78.3	[70.5; 92.5]	**<0.001**	**<0.001**
RV-ESV (indexed, mL/m²)		42.7	[35.8; 49.0]	35.0	[30.0; 41.8]	**0.030**	**0.007**
RV-EF (%)		56.0	[52.2; 60.4]	55.4	[51.9; 58.7]	0.467	0.448
RV-GLS (-%)		24.0	[20.8; 26.5]	22.7	[19.9; 25.5]	0.807	0.806
RV-SV (indexed, mL/m²)		53.7	[50.5; 57.3]	45.6	[41.1; 48.6]	**<0.001**	**0.004**
LA volume		74	[59; 80]	56	[43; 67]	**0.012**	**0.018**
LA longitudinal strain (%)		40.0	[30.9; 46.8]	33.7	[28.8; 42.8]	0.192	0.154
RA volume		83	[73; 96]	67	[59; 80]	**0.024**	**0.009**
RA longitudinal strain (%)		36.0	[27.1; 51.1]	42.3	[31.4; 48.3]	0.579	0.560
Global T1 times (ms, 1.5T)		1092	[1070; 1098]	1009	[992; 1022]	**0.042**	0.159
Global T1 times (ms, 3T)		1232	[1212; 1246]	1232	[1204; 1256]	0.544	0.499
Global T2 times (ms, 1.5T)		49	[48; 50]	47	[46; 48]	0.157	0.079
Global T2 times (ms, 3T)		41	[39; 42]	40	[38; 42]	0.194	0.179

*LV* left ventricular, *EDV* end-diastolic volume, *ESV* end-systolic volume, *EF* ejection fraction, *GLS* global longitudinal strain, *SV* stroke volume, *RV* right ventricular, *LA* left atrial, *RA* right atrial, *COVID* coronavirus disease, *CMR* cardiovascular magnetic resonance

The local reference values for the mapping sequences are:

1.5T: 1010 ms [910–1110] for T1 and 48 ms [44–52] for T2

3T: 1180 ms [1116–1244] for T1 and 38 ms [32–44] for T2

Values are given as median (Interquartile range).

Significant group differences are highlighted in bold.

* Adjusted for age, sex, hypertension, and asthma

### 3.3 Vasoactive breathing maneuver

**Fig. 3:**
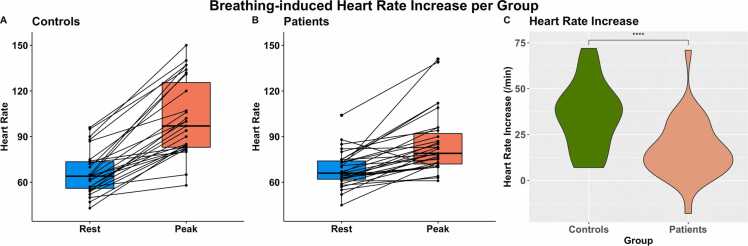
Individual heart rate response in controls (A) and cases (B) before and during a 60-s metronome-paced hyperventilation. (C) Displays the heart rate increase between groups. The heart rate response was blunted (p < 0.001) in cases as opposed to controls

**Fig. 4:**
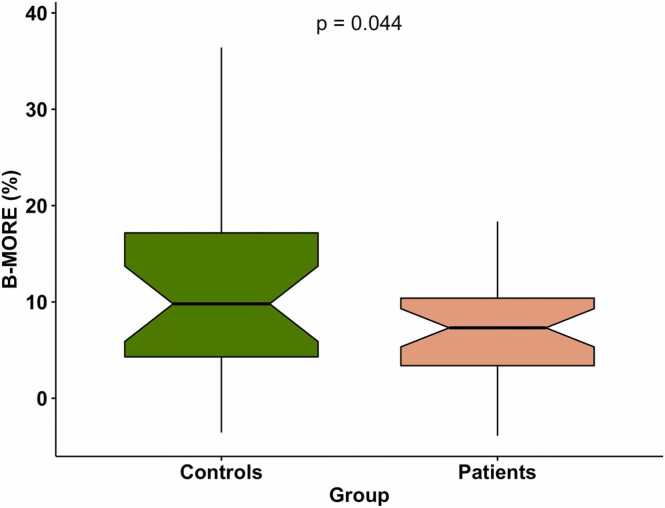
Breathing-induced myocardial oxygenation reserve (B-MORE) in cases and controls

At rest, patients and controls had a normal resting heart rate as well as inconspicuous oxygen saturation and blood pressure. During metronome-paced hyperventilation, a similar portion of controls and patients experienced temporary side effects (55.6% vs 64.5%). Vital parameters and side effects are detailed in Table 4. Patient compliance, breathing depth, and breathing rate were excellent (26/31 patients [83.9%]; 23/27 controls [85.2%]) or good (5/31 [16.1%]; 4/27 [14.8%]) in all participants. Field strength was not associated with B-MORE results (p = 1.0). In contrast, the heart rate response was significantly different between patients and controls (p < 0.001) with an increase of 35/min [21; 45] in controls and 15/min [7; 26] in patients, illustrated in Fig. 3 and Table 4. After adjustment for age, sex, arterial hypertension, and asthma, the heart rate response remained significant (p < 0.001), while only age (p = 0.021) and asthma (p = 0.036) revealed a significant correlation with the heart rate response (p > 0.05 for the rest). The B-MORE was 9.8% in controls [4.3; 17.2] and 7.3% in patients [3.4; 10.4] with a significant difference between the groups (p = 0.044). After adjusting for age, sex, arterial hypertension, and asthma, B-MORE remained significant (p = 0.049), while none of the confounders were significantly associated with B-MORE (p > 0.05 for all). Fig. 4 and Table 4 show B-MORE results for both groups and detail unadjusted and adjusted group differences. Both heart rate response and B-MORE were not correlated with NTproBNP levels, presence and severity of shortness of breath, left ventricular EF, volume, or GLS (p > 0.05 for all). In controls, neither heart rate response nor B-MORE was different between athletic and non-athletic participants (p = 0.827; p = 0.542). In a subgroup analysis of the patient group, there was no statistical difference in heart rate response (p = 0.372) or B-MORE (p = 0.834) between those after COVID illness versus those after vaccination. The full subgroup analysis can be found in Supplementary Table 1.

**Table 4:** Results from the hyperventilation stress between cases and controls.

						Group difference	
		Controls		Long COVID		Unadjusted	Adjusted*
		n = 27		n = 31		p =	
Length of breath-hold at expiration (s)							
	≥60	23	85.2%	26	83.9%	0.551	0.318
	≥45	2	7.4%	1	3.2%		
	≥35	2	7.4%	4	12.9%		
Side effects during hyperventilation		15	55.6%	20	64.5%	1.0	NA
	Tingling sensation	10	37.0%	11	35.5%		
	Dizziness	5	18.5%	5	16.1%		
	Other†	6	22.2%	3	9.7%		
Blood pressure at rest (mmHg)							
	Systolic	131	[120; 141]	125	[116; 135]	0.080	0.066
	Diastolic	80	[72; 83]	77	[72; 84]	0.380	0.388
Oxygen saturation at rest		98	[95; 99]	98	[96; 99]	0.539	0.646
HR at rest (/min)		64	[56; 74]	66	[62; 74]	0.578	0.575
Peak HR during hyperventilation (/min)		97	[83; 125]	79	[72; 92]	**0.005**	**0.004**
Heart rate difference		35	[21; 45]	15.0	[7; 26]	**<0.001**	**<0.001**
B-MORE (%)		9.8	[4.3; 17.2]	7.3	[3.4; 10.4]	**0.044**	**0.049**

*HR* heart rate, *B-MORE* Breathing-induced myocardial oxygenation reserve, *COVID* coronavirus disease

Significant group differences are highlighted in bold.

Continous values are given as median (Interquartile range). Binary values are given as percentages with numerator and denominator.

* Adjusted for age, sex, hypertension, and asthma

† Headache, dry mouth, palpitations. No serious side effects occurred. All side effects ceased within 30 s

## 4 Discussion

This present study is the first to show a potential benefit of using OS-CMR and breathing maneuvers in the assessment of long COVID patients. In contrast to a group of controls indifferent concerning age, gender, body mass index, and cardiovascular risk factors, the hyperventilation-induced heart rate increase was blunted in long COVID cases (15/min vs 35/min, p < 0.001). Additionally, OS-CMR revealed a significant difference in B-MORE between controls (B-MORE 9.8%) and cases (B-MORE 7.3%, p = 0.044), which remained significant after adjusting for age, sex, hypertension, and asthma (p = 0.049). Taken together, these results might suggest an impaired vascular responsiveness and presence of endothelial dysfunction.

CMR may be the current gold standard to assess structural pathologies and has proven valuable in diagnosing acute myocardial inflammation following COVID-19 infection or vaccination [25], [26], [27], [28]. However, its role in the assessment of long-term myocardial changes is less established. In the literature, a mild vaccine- or infection-related long-term myocardial injury has been described [5], [29], [30], [31]. However, assessed parameters such as LV-EF, GLS, and T1/T2 times were still within normal range and detected changes did not correlate with clinical symptoms [5], [7], [29], [30]. Similarly, the standard CMR sequences in the present study did not reveal any significant cardiac pathologies. Consistent with previous literature, indexed stroke volumes of patients were lower when compared with controls, but still within normal range [30]. It remains unlikely that these deviations in stroke volume and previously found mild myocardial tissue abnormalities alone are responsible for the clinical presentation of long COVID, leaving the pathophysiology of long COVID unanswered. Here, our study may provide a new link pointing toward the endothelium as a critical structure in the development of long COVID. Hyperventilation is a natural stressor inducing an endothelium-dependent vasoconstriction mediated through low CO_2_ levels, while the post-hyperventilation breath-hold leads to an increase in CO_2_ and subsequent vasodilation [22]. Coronary vasodilation in healthy individuals results in a “luxury perfusion” with increased coronary blood flow without an associated increase of demand, which decreases the proportion of deoxygenated hemoglobin in the myocardium. In OS-CMR images, this leads to a myocardial signal intensity increase, which can be semi-quantitatively expressed with the B-MORE [15]. Hyperventilation, however, also activates the neuro-humoral axis and increases the rate-pressure product, which increases demand. This triggers a heart rate increase that is reduced in patients with heart disease [32]. Since both B-MORE and heart rate increase are diminished in long COVID patients when compared to controls in our study, a crucial role of the endothelium seems likely. This is in accordance with extensive literature on the pathophysiology of long COVID, although this study is the first to apply this to the heart and test it clinically [11], [12], [13], [33], [34]. The inability to adequately react to external or internal stress on top of a decreased stroke volume despite a structurally inconspicuous heart might explain many of the symptoms described by long COVID patients [23], [35]. This study is only a first step toward a potential implementation of OS-CMR with vasoactive breathing maneuvers into the standard assessment of long COVID patients, as the protocol currently still faces obstacles. Specifically, the overlap between cases and controls leaves a level of uncertainty for the individual patient. However, previous studies have demonstrated that the heterogeneity of myocardial oxygenation might play a significant role [19]. Normal (or even elevated) segments can coexist with pathologically reduced ones, potentially masking abnormalities in the global signal despite high levels of abnormalities. Furthermore, the introduction of machine learning-enhanced analysis of the whole cardiac cycle may significantly improve the signal-to-noise ratio. The measurements of B-MORE in the control group in the present study were comparable to previous measurements of healthy individuals in other studies [15], [17]. Interestingly, a recent study did not find any perfusion deficits in a cohort of long COVID patients, which might seem in contrast to our study’s findings [36]. However, previous studies comparing both perfusion and oxygenation in the same patients indicated that these are overlapping but distinct phenomena [37], [38].

## 5 Limitations

Our study has several limitations. First, as a hypothesis-generating case-control study, the case number is limited, and results may not apply to other populations. Specifically, the indifference between the post vaccination and post COVID subgroups might not hold in a larger study group. Future studies and larger case numbers are needed to confirm the described observations and to apply them to other organ systems as well. Second, it remains possible that the differences between the two groups were caused by different levels of compliance with the breathing maneuvers. To address this, all participants were instructed to follow a highly standardized protocol and practice hyperventilation. Furthermore, patient compliance was ensured both visually and automatically and the derived rating scale revealed an excellent or good compliance in all cases. Since the breath-hold was at expiration, its length of >35 s in all participants and ≥60 s in 83.9% of the participants points toward a significant effect of hyperventilation. Otherwise, a breath-hold of that length would be hardly achievable at expiration. Ultimately, the rate of hyperventilation-induced side effects did not differ between patients and controls. Thus, a significant bias is highly unlikely. Third, an objective assessment of physical activity of both patients and controls would have been valuable for correlating with the study’s findings. Fourth, the analysis of late gadolinium enhancement images would have certainly been of interest to achieve better comparability to previous studies. However, we believe that the risk of missing these mild changes of unknown clinical significance is outweighed by the benefit of the fast and needle-free CMR protocol that saves both time and resources. Fifth, an underlying lung pathology may have contributed to the results as it may limit the peak oxygen uptake. However, this cannot explain the blunted heart rate response and patients did not have any known lung comorbidity. Sixth, the inclusion of both patients post COVID vaccination and post COVID illness into one study group assumes that both entities are clinically comparable. However, research especially for the pathophysiology of symptoms after COVID vaccination is scarce and the disease is not yet well understood [4]. In this study, however, apart from length of symptoms, a detailed subgroup analysis (Supplementary Table 1) did not reveal any statistical differences between both subgroups, neither concerning clinical presentation nor cardiac biomarkers, standard CMR, or hyperventilation stress OS-CMR.

## 6 Conclusions

In conclusion, the current study introduces breathing maneuvers and OS-CMR as new tools in the assessment of long COVID patients that may extend the diagnostic capabilities of CMR and highlight a potential role of the endothelium in the pathophysiology of long COVID. The results indicate an impaired hemodynamic and myocardial oxygenation responsiveness, possibly reflecting an altered coronary vasoreactivity and a direct impact of COVID-19 on coronary vascular integrity. However, this should be confirmed in larger trials and correlated to symptoms and objectifiable functional capacity.

## Funding

For the analysis of the OS-CMR images, the authors were provided with one free research license by Circle Cardiovascular Imaging. L.D.W. was supported by the Rotation Grand (D.10021788) of the 10.13039/100010447DZHK (German Centre for Cardiovascular Research). Circle Cardiovascular Imaging and the DZHK did not have any influence on design, analysis, or interpretation of the study.

## Author contributions

**Lukas D. Weberling:** Writing – review & editing, Writing – original draft, Visualization, Supervision, Project administration, Methodology, Investigation, Funding acquisition, Formal analysis, Data curation, Conceptualization. **Matthias G. Friedrich:** Writing – review & editing, Supervision, Software. **Elizabeth Hillier:** Writing – original draft, Supervision, Methodology. **Norbert Frey:** Writing – review & editing, Resources. **Marc Zahlten:** Writing – review & editing, Methodology, Investigation, Data curation. **Henning Steen:** Writing – review & editing, Writing – original draft, Supervision, Resources, Project administration, Investigation, Funding acquisition, Conceptualization. **Florian André:** Writing – review & editing, Supervision.

## Ethics approval and consent

This study was approved by the ethical committee of our university (S-361/2022) and carried out in accordance with the Declaration of Helsinki. Written informed consent was given by all patients and controls.

## Declaration of competing interests

The authors declare the following financial interests/personal relationships which may be considered as potential competing interests: Lukas D. Weberling reports equipment, drugs, or supplies were provided by Circle Cardiovascular Imaging Inc. Lukas D. Weberling reports article publishing charges were provided by German Research Foundation. Lukas D. Weberling reports financial support was provided by German Center for Cardiovascular Disease. Matthias G. Friedrich and Elizabeth Hillier report a relationship with Area 19 Medical Inc. that includes consulting or advisory. The other authors declare that they have no known competing financial interests or personal relationships that could have appeared to influence the work reported in this paper.

## Data Availability

The datasets used and analyzed during the current study are available from the corresponding author on reasonable request.
