# Supplementary material for: Abnormal coronary vascular response in patients with long COVID syndrome – a case‐control study using oxygenation‐sensitive cardiovascular magnetic resonance
Source: J Cardiovasc Magn Reson. 2025 Apr 2;27(1):101890. doi: 10.1016/j.jocmr.2025.101890 (PMC12182812; doi:10.1016/j.jocmr.2025.101890)
Supplement: Supplementary file 1 — Supplementary material [file mmc1.docx]

**Supplementary Material**

**Supplementary Table 1**: Subgroup analysis of the patients. Portrayal of differences between those post COVID illness versus those post COVID vaccination. NA = not available since no patients of this group were scanned at that field strength.
